# High Viral Load of Human Bocavirus Correlates with Duration of Wheezing in Children with Severe Lower Respiratory Tract Infection

**DOI:** 10.1371/journal.pone.0034353

**Published:** 2012-03-30

**Authors:** Yu Deng, Xiaoyang Gu, Xiaodong Zhao, Jian Luo, Zhengxiu Luo, Lijia Wang, Zhou Fu, Xiqiang Yang, Enmei Liu

**Affiliations:** 1 Department of Respiratory Medicine, Children's Hospital, Chongqing Medical University, Chongqing, People's Republic of China; 2 Department of Nephrology and Immunology Medicine, Children's Hospital, Chongqing Medical University, Chongqing, People's Republic of China; 3 Laboratory of Children's Respiratory Medicine, Ministry of Education Key Laboratory of Child Development and Disorders, Chongqing Medical University, Chongqing, People's Republic of China; 4 Paediatric unit of the Bethune International Peace Hospital of the PLA, Shijiazhuang Hebei Province, People's Republic of China; University of Liverpool, United Kingdom

## Abstract

**Background:**

Human bocavirus (HBoV) is a newly discovered parvovirus and increasing evidences are available to support its role as an etiologic agent in lower respiratory tract infection (LRTI). The objective of this study is to assess the impact of HBoV viral load on clinical characteristics in children who were HBoV positive and suffered severe LRTI.

**Methods:**

Lower respiratory tract aspirates from 186 hospitalized children with severe LRTI were obtained by bronchoscopy. HBoVs were detected by real-time PCR and other 10 infectious agents were examined using PCR and/or direct fluorescent assay.

**Results:**

Thirty-one patients (24.6%) were tested positive for HBoV in the respiratory tract aspirates. Fifteen samples had a high viral load (>10^4^ copies/mL) and the other sixteen samples had a low viral load (<10^4^ copies/mL). The duration of presented wheezing and hospitalization was longer in children with high viral load of HBoV than that in children with low viral load. The days of wheezing showed a correlation with viral load of HBoV.

**Conclusion:**

We confirmed that HBoV was frequently detected in patients with severe LRTI. Wheezing was one of the most common symptoms presented by patients with positive HBoV. A high HBoV viral load could be an etiologic agent for LRTI, which led to more severe lower respiratory tract symptom, longer duration of wheezing and hospitalization.

## Introduction

Human bocavirus (HBoV) is a newly discovered parvovirus that was first identified in Sweden from pooled nasopharyngeal aspirate specimens by large-scale molecular virus screening [1]. Increasing evidences are emerging to support its role as an etiologic agent in lower respiratory tract infection (LRTI) [2], [3], [4], [5].

HBoV is frequently detected in patients with acute respiratory tract infection 6,7,8, including in those who are having wheezing, croup, cough, rhinorrhea and fever [2], [9]. A number of epidemiological and clinical investigations have been conducted to assess HBoV- related illness; its clinical features have been reported, which resembled those of respiratory syncytial virus (RSV) and human metapneumovirus (hMPV) infections [10]. The most frequent clinical diagnoses associated with respiratory HBoV are general upper respiratory tract infections, bronchiolitis, pneumonia, bronchitis and asthma exacerbation [2]. Nevertheless, few data are available related to physical characteristics and clinical severity for children with HBoV-positive LRTIs [7].

In this study we aimed to assess the relationship between the HBoV viral load in respiratory tract and clinical characteristics. To achieve this goal, we conducted the study to compare the clinical characteristics, cytokine/chemokine data in respiratory tract aspirates between two groups of children who had HBoV infection with either high or low viral load.

## Methods

### Research subjects and respiratory tract aspirates collection

The study population was hospitalized (in the Department of Respiratory Medicine, Children's Hospital, Chongqing Medical University) and underwent bronchoscopy. The study was conducted from April 2006 to March 2009. A total of 186 sequential respiratory tract aspirate samples were collected. Informed consent was properly obtained from each patient's guardians. The informed consent was written and signed by guardian before bronchoscopy. This study was approved by the Ethical Committee of Chongqing Medical University.

On admission, the medical history and physical examination of the children were properly obtained and recorded systematically. In this study, we defined LRTI as having following characteristics: presence of wheezing, crackling rales, dyspnea, and/or obstruction of the airways, with or without fever, and presence of upper airway symptoms characterized by rhinorrhea and bronchitic cough. All patients with suspected LRTI underwent chest radiography. Pneumonia was defined as the presence of focal infiltration or lung consolidation presented in chest radiography.

### Respiratory tract aspirates preparation

After respiratory tract aspirates were collected, the aspirates were gently mixed with Pasteur pipette and revolve for a while until they were evenly mixed, then 0.5 ml of each aspirate was transferred to another tube for cytokine analysis. Four volumes of 0.1% dithiothreitol was added to the 0.5 ml respiratory tract aspirates, then gently mixed with Pasteur pipette, votexed 15 seconds and shook 15 minutes on bench rocker. The suspension was subsequently filtered through 48 µm nylon gauze to remove mucus and debris without removing any cells, and then it was centrifuged at 1500 rpm for 10 minutes. The cell free supernatant was stored at −80°C for cytokine assay. The cell pellet was resuspended for viral antigen detection using direct fluorescent assay inmediately. The rest of aspirates were stored at −80°C before virus detection by polymerase chain reaction (PCR) assay.

### Real-time PCR for HBoV

Nucleic acids were extracted from 200 µL of the aspirates using the QIAamp DNA mini kit (Qiagen, USA) and the samples were eluted in 200 µL of RNasefree water. The PCR assay targeted the NP-1 gene of HBoV. The 20-µL amplification reaction contained 5 µL of sample DNA, 10 µL of TaqMan universal PCR master mix (PE Applied Biosystems), 0.1 µL of bovine serum albumin (20 mg/mL), 300 nmol/L each primer (Boca-forward, GGA AGA GAC ACT GGC AGA CAA; and Boca-reverse, GGG TGT TCC TGA TGA TAT GAG C), and 150 nmol/L Boca probe (FAM-CTG CGG CTC CTG CTC CTG TGA T-TAMRA). Amplification was performed on a LightCycler 1.2 instrument (Roche) with the following settings: 95°C for 10 min, 50 cycles of 95°C for 30 sec, and 60°C for 1 min. For standardizing the quantification, a plasmid (NPSC3.1) containing the HBoV NP-1 gene was used in serial dilutions covering a range of 6 logs. The criteria for a positive reaction were defined as the cycle threshold <40 cycles and fluorescence count >0.5. The minimum genome viral load that would allow reproducible quantification was 10 copies per reaction, which corresponds to 500 copies/mL of respiratory tract aspirates. The analyses were performed at a diagnostic laboratory, where rigorous measures were implemented to prevent contamination.

### Means of diagnosis for other viruses

Viral antigens were analyzed for adenovirus (ADV), influenza virus A and B (Flu-A and B), parainfluenza virus 1, 2 and 3 (PIV 1–3) and RSV using direct fluorescent assay (D3 direct fluorescent assay RESPIRATORY VIRUSES SCREENING & ID KIT, DIAGNOSTIC HYBRIDS, Germany); PCR was used for the detection of ADV; reverse transcription-PCR was used for the detection of rhinovirus (RV), RSV, human coronaviruses OC43 (HCoV), Flu-A and B, PIV 1–3 and hMPV. DNA was extracted from 200 µL of the aspirates using the QIAamp DNA mini kit (Qiagen, USA) and was eluted in 200 µL AE buffer. RNA was extracted from 140 µL of the aspirates using the QIAamp Virus RNA Mini kit (Qiagen, USA), and samples were eluted in 60 µL of RNase-free water. Reverse transcription of 0.5 mg of each RNA was performed in a final reaction of 10 µL that contained 25-pmol Oligo dT primer, 50 pmol random 6-mers, 5× PrimeScript Buffer and 0.5 µL PrimeScript RT Enzyme Mix (TaKaRa, Japan). Amplification was performed with a Bio-Rad thermal cycler, using commercially available master mixes (Promega, USA) and standard protocols. All PCR assays were performed using 2 µl of cDNA/DNA and 1 mM of each primer. In addition, the primer sequences, PCR protocols and other viral detection methods are described elsewhere [11]. The unclassified RVs were identified as RVs. A patient was considered to be positive for the virus only if having at least one positive result from the two methods.

### Bacterial isolation

Bacterial/yeast diagnostic samples were processed immediately in a qualitative and semi-quantitative ways using standard microbiological methods [12], [13]. For all samples, macroscopically distinct colonies were isolated in pure culture and standard methods for identification, typing, and sensitivity patterns were used.

### Cytokine/chemokine analysis

By following the instructions, respiratory tract aspirates were tested for interleukin-6 (IL-6), interleukin-8 (IL-8) and interleukin-10 (IL-10) by enzyme linked immunosorbent assay (ELISA) (BD Pharmingen, USA). Each sample was measured in duplicate using VARIOSKAN FLASH (Thermo) by following manufacturer's instructions. The concentration of each cytokine was determined by interpolation from the corresponding standard curve. When the values were below the detection threshold, the minimum detectable level was assigned.

### Statistical analysis

Data on the descriptive characteristics of the patients were analyzed by χ2, Fisher's exact test. Non-parametric tests and median values were used for those variables, because they were not normally distributed, such as cytokine/chemokine concentrations. Correlations were made with Spearman's two-tailed rank correlation in addition to Pearson's linear regression two-tailed analysis. For all outcomes and relations between variables, differences between groups were considered statistically significant at P<0.05. SPSS (version 17.0) was used for all analysis.

## Results

### Frequency and distribution of HBoV detection

The patient demographics and clinical information were summarized in Table 1. HBoV DNA was detected by PCR in 31 out of 186 patient's samples (24.6%). All samples were also tested for the other respiratory viruses, including RSV (32.8%), RV (22.4%), hMPV (2.4%), FLU (20%), HPIV (44%), HCoV (1.6%) and ADV (4.8%). Among all 31 HBoV-positive samples, 12 samples (38.71%) were only being detected for HBoV, while in other 19 samples there were one more virus were being detected besides HBoV. It was noticed that the most common coinfection viruses with HBoV were RSV (in 4 children), RV (in 3 children) and ADV (in 2 children).

**Table 1:** Demographic and clinical characteristics of patients (n = 186).

Clinical data	n (*%*)
Mean age (Months ± SEM)	23.41±2.56
Male	112 (60.22)
Median hospital length of stay (median, IQR) (days)	9 (2–31)
Chest radiograph	
Any abnormality	150 (80.65)
Multifocal infiltrates or consolidation	16 (10.67)
Diffuse infiltrates	65 (43.33)
Hyperinflation	17 (6.00)
Atelectasis	21 (14)
Interstitial abnormality	39 (26.00)
Clinical diagnosis	
Bronchial pneumonia	15 (8.06)
Interstitial lung diseases	109 (58.60)
Asthma	39 (20.97)
Bronchiolitis obliterans	6 (3.23)
Others	17 (9.14)

HBoV DNA was detected all year round, seasonal distribution of HBoV was noted during October through March. The period from January to March was the seasonal peak that overlapped with the period of RSV peak, which may explained that why RSV was being the most common co-infection virus with HBoV.

### Clinical data of patients with or without HBoV

For the purpose of the analysis, the patients were divided into two groups: those with HBoV infection and those without HBoV infection. The most frequent respiratory symptoms/signs (present in >50%) seen in HBoV-positive patients were cough and wheezing. Significantly high frequency of wheezing (90.322% vs. 72.903%, p = 0.04) and dyspnea (41.935% vs. 13.548%, p = 0.001) were noted in 31 HBoV-positive patients, in comparison with 155 HBoV-negative patients. (Table 2). Furthermore a significant difference was noted in the length of hospital stay between HBoV- positive and HBoV-negative patients (Table 2). In general, the clinical findings were more severe in patients with HBoV infection.

**Table 2:** Clinical features of HBoV-positive patients.

Clinical data	HBoV-positive samples (n = 31)	HBoV-negative samples (n = 155)	P
Cough	31 (100%)	155 (100%)	1
Wheezing	28 (90.32%)	113 (72.90%)	0.040*
Fever	13 (41.94%)	51 (32.90%)	0.408
Tachypnea	20 (64.52%)	79 (50.97%)	0.237
Dyspnea	13 (41.94%)	21 (13.55%)	0.001*
Rhinorrhea	6 (19.36%)	19 (12.26%)	0.384
Cyanosis	12 (38.71%)	61 (39.36%)	1
Duration of hospitalization (days) (mean±SEM)	12.10±1.101	9.903±0.456	0.0292**

* Calculated using the fisher's exact test. P<0.05 was considered to be significantly different between each other.

** Calculated using the Mann-Whitney U test. P<0.05 was considered to be significantly different between each other.

### Clinical characteristics of patients who had positive HBoV with or without co-infection with other viruses or bacteria

The clinical characteristics of children who had detectable HBoV with or without co-infection with other viruses or bacteria were shown in Table 3 and Table 4. In table 3, the two groups were similar in all clinical manifestations, duration of hospitalization and wheezing.

**Table 3:** Clinical characteristic of children who had HBoV detection with or without detection of other viruses.

Clinical data	HBoV sole (n = 12)	HBoV coinfection (n = 19)	P
Cough	12 (100%)	19 (100%)	1.000
Wheezing	12 (100%)	17 (89.47%)	0.510
Fever	5 (41.67%)	9 (47.37%)	1.000
Tachypnea	9 (75.00%)	11 (57.89%)	0.452
Dyspnea	9 (41.94%)	14 (13.55%)	1.000
Rhinorrhea	2 (16.67%)	4 (21.05%)	1.000
Cyanosis	11 (38.71%)	16 (39.36%)	1.000
Duration of hospitalization (days) (mean±SEM)	11.92±1.852	12.21±1.403	0.855
Duration of presented wheezing (days) (mean±SEM)	23.33±2.23	21.42±2.72	0.9515

* Calculated using the fisher's exact test. P<0.05 was considered to be significantly different between each other.

** Calculated using the Mann-Whitney U test. P<0.05 was considered to be significantly different between each other.

**Table 4:** Clinical characteristic of children who had HBoV detection with or without detection of bacteria.

Clinical data	HBoV without bacteria detection (n = 10)	HBoV with bacteria detection (n = 21)	P
Cough	10 (100%)	21 (100%)	1.000
Wheezing	8 (80%)	20 (95.24%)	0.237
Fever	5 (50%)	8 (38.10%)	0.701
Tachypnea	6 (60%)	14 (66.67%)	1.000
Dyspnea	3 (30%)	5 (23.81%)	1.000
Rhinorrhea	3 (30%)	4 (19.04%)	0.681
Cyanosis	4 (40%)	8 (38.10%)	1.000
Duration of hospitalization (days) (mean±SEM)	10.00±1.033	13.10±1.516	0.236
Duration of presented wheezing (days) (mean±SEM)	18.60±4.47	23.86±1.714	0.410

* Calculated using the fisher's exact test. P<0.05 was considered to be significantly different between each other.

** Calculated using the Mann-Whitney U test. P<0.05 was considered to be significantly different between each other.

Among all HBoV-positive samples, 67.74% were also positive for bacteria. No significant difference was found in whether it was associated with bacteria or not (Table 4). Therefore, our findings did not support the existence of significant interactions between HBoV and other respiratory viruses or bacteria. This finding clearly suggested the pathogenic potential of HBoV in young children with LRTI.

### Quantitative analysis of HBoV DNA in the respiratory aspirates

**Figure 1:**
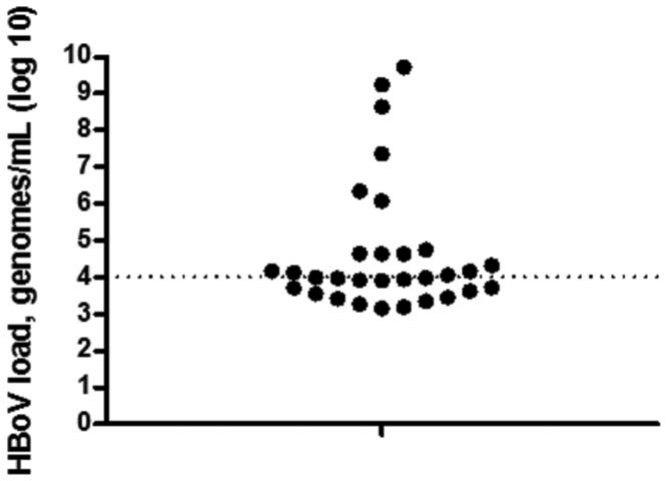
Distribution of human bocavirus (HBoV) viral loads. Thirty one respiratory aspirate samples were tested positive for HBoV. Each sample is represented by a single dot. Viral loads in the nasopharyngeal aspirates ranged from <500 to 1×10^9^ copies per mL of sample material. The dotted line indicates the cutoff between the high (>10^4^ copies/mL) and low (<10^4^ copies/mL) HBoV viral load groups.

Absolute quantification of DNA by qPCR revealed that the HBoV viral load in HBoV-positive patients varied very broadly, ranged from <500 to 1×10^9^ copies per mL of sample material, with median value of 9912 (figure 1). The median viral load which was found in patients with sole HBoV infection was not higher than those who had co-infection with other respiratory viruses (p = 0.2517). A recent review suggested if serum samples were not available for PCR, and then the probable next best option was the quantitative PCR with a cutoff of >10^4^ copies/mL of respiratory tract secretions [14]. Several previous studies reported that increased serum HBoV IgM and IgG had been found in most of patients who had high HBoV load (>10^4^ copies/mL) in respiratory tract secretions, which was associated with respiratory symptoms [2], [15], [16]. Therefore the HBoV-positive samples were divided into 2 non-overlapping populations: one group of 15 samples with a high viral load (>10^4^ copies/mL) and one group of 16 samples with a low viral load (<10^4^ copies/mL).

### Clinical characteristics of patients with high HBoV viral load

High HBoV viral load was detected in 15 patients. The comparisons of demographics and clinical data among patients who were shedding high HBoV (>10^4^ copies/mL of respiratory tract aspirates) and those who were shedding low HBoV were shown in table 5. In HBoV-positive patients, the distributions of age and gender were not different between the two groups. Almost all of HBoV-positive patients presented cough and wheezing. Non-specific lower respiratory tract symptoms, such as fever and rhinorrhea, had no significant correlation with HBoV viral load. However, the symptoms observed in patients with serious LRTIs, like tachypnea, dyspnea and cyanosis, were presented more frequently in children with high HBoV viral loads, and the mean duration of hospitalization in these children was 14.67±1.93 days, which was significantly longer than in children with low HBoV viral load.

**Table 5:** Clinical data of patients with different HBoV viral load.

Clinical data	HBoV-positive patients, viral load		P
	<10^4^ copies/mL (n = 16)	≥10^4^ copies/mL (n = 15)	
Masculine gender	10(62.5%)	10(66.7%)	1
Age (median of months)	6.5	10	0.178
Cough	16(100%)	15(100%)	1
Wheezing	14(87.5%)	14(93.33%)	1
Fever	8(50%)	5(33.3%)	0.473
Tachypnea	7(43.75%)	13(86.67%)	0.023*
Dyspnea	3(18.75%)	10(66.7)	0.011*
Rhinorrhea	4(25%)	2(13.33%)	0.654
Cyanosis	3(18.75%)	9(60%)	0.029
Duration of hospitalization (days) (mean±SEM)	8.75±0.91	14.67±1.93	0.0082**
Duration of presented wheezing (days) (mean±SEM)	26.43+2.27	20.47+2.115	0.0274**

* Calculated using the fisher's exact test. P<0.05 was considered to be significantly different between each other.

** Calculated using the Mann-Whitney U test. P<0.05 was considered to be significantly different between each other.

### Cytokine/chemokine concentration

Cytokine/chemokine concentrations of the respiratory tract aspirates from patients with HBoV infection were shown in Table 6. In patients with high HBoV viral load, IL-8 level was found higher than those who had low HBoV viral load. The IL-6 and IL-10 levels were very similar between the two groups.

**Table 6:** Cytokine/chemokine concentrations of respiratory tract aspirates in children with different HBoV viral load.

Cytokine/chemokine (pg/mL)	<10^4^ copies/mL (n = 16)	≥10^4^ copies/mL (n = 15)	P
Interleukin-8	1989±435.6	405.4±162.1	0.0015**
Interleukin-6	21.77±16.79	49.71±27.7	0.6597
Interleukin-10	36.23±10.92	42.64±8.416	0.7685

** Calculated using the Mann-Whitney U test. P<0.05 was considered to be significantly different between each other.

In order to identify whether the potential co-infections would influence the cytokine production and the biological significance of IL-8, IL-6 and IL-10, we compared IL-8, IL-6 and IL-10 productions between patients with only HBoV detection, and patients with HBoV and other viruses or bacteria detection, no significant differences were found (table 7 and table 8).

**Table 7:** Cytokine/chemokine concentrations of respiratory tract aspirates in children who had HBoV detection with or without detection of other viruses.

Cytokine/chemokine (pg/mL)	HBoV sole (n = 12)	HBoV coinfection (n = 19)	P
Interleukin-8	1804±711.3	2599±738.3	0.488
Interleukin-6	30.30±5.988	49.81±13.94	0.533
Interleukin-10	40.35±13.21	53.59±7.858	0.7371

** Calculated using the Mann-Whitney U test. P<0.05 was considered to be significantly different between each other.

**Table 8:** Cytokine/chemokine concentrations of respiratory tract aspirates in children who had HBoV detection with or without detection of bacteria.

Cytokine/chemokine (pg/mL)	HBoV without bacteria detection (n = 10)	HBoV with bacteria detection (n = 21)	P
Interleukin-8	2413±1110	2219±611.8	0.977
Interleukin-6	31.50±8.051	46.03±11.76	0.687
Interleukin-10	52.63±14.90	47.45±7.389	0.941

** Calculated using the Mann-Whitney U test. P<0.05 was considered to be significantly different between each other.

### Association of HBoV in respiratory tract aspirates, duration of hospitalization and days of wheezing

**Figure 2:**
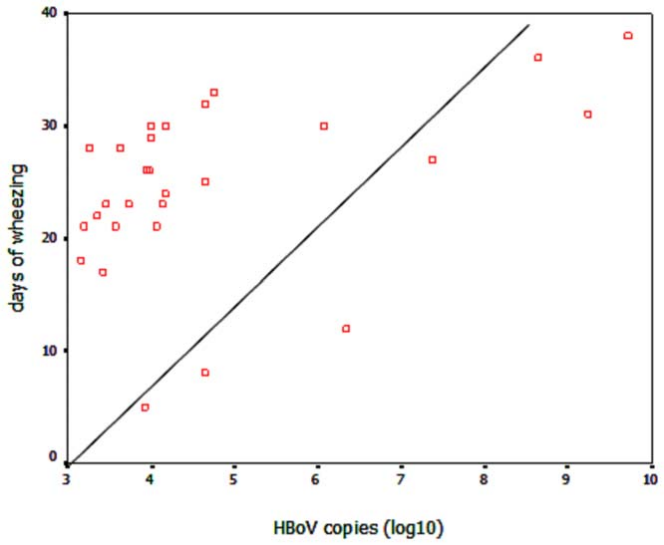
Correlation between HBoV viral load and days of wheezing. HBoV viral load was correlated to the days of presented wheezing. Correlations were made with Spearman's two-tailed rank correlation in addition to Pearson's linear regression two-tailed analysis. Correlation coefficient = 0.538, P = 0.003.

Longer duration of hospitalization and days of wheezing were found among children with high HBoV loads (table 5). Then we looked at the correlation between viral loads and duration of hospital stay and days of wheezing, we searched for additional evidence of etiology by studying whether the viral load of HBoV was unrelated or correlated to the days of hospitalization and presented wheezing. And we found that the days of wheezing showed a direct correlation with viral load (Correlation coefficient = 0.538. P = 0.003) (Figure 2).

## Discussion

In this study, 24.6% of patients with serious lower respiratory tract illness were detected having HBoV in their respiratory tract aspirates. We confirmed that HBoV was frequently detected in children with severe LRTIs. Wheezing was one of the most common symptoms presented by HBoV-positive patient. HBoV at a high load could be an etiologic agent for LRTIs, which led to more severe lower respiratory tract symptom, longer hospitalization, and longer duration of wheezing.

HBoVs have been detected worldwide, low HBoV viral load was usually more frequently detected than high HBoV viral load in children [17], [18], [19], its role as a pathogen in respiratory tract was often questioned. However, HBoV at a high load were clearly suggested for being an etiologic agent for respiratory tract disease [2]. In our study, fifteen out of thirty-one HBoV-positive patients who had high HBoV viral loads, presented acute primary infection. Symptoms, duration of hospitalization were compared among patients who had LRTI with or without HBoV infection. We found that in patients of LRTIs with HBoV infection needed longer hospital stay. These findings suggested that HBoV was one of the common pathogens for children with serious LRTIs.

Although, increasing number of studies suggested the pathogenic potential of HBoV in young children with respiratory tract infections, however HBoVs were frequently detected in asymptomatic children's respiratory tract secretions, which may raise some justifiable concerns for etiology [1], [20]–[29]. The variation of HBoV detection in these studies was likely attributable to multiple factors, such as patient's age, season, geographic location, primer sensitivity, laboratory technique, true variation in incidence of HBoV, and methods used in sample collection. Some studies suggested that persistent virus shedding was therefore being a reasonable explanation for the high occurrence of HBoV in healthy subjects [20]. Moreover failure for follow-up on HBoV-positive asymptomatic children also led to high occurrence of HBoV detection in asymptomatic children. Recently, an *in vitro* study of HBoV infection in pseudostratified epithelial cells was established to confirm the virus uptake, transcription, and replication [30]. This study supports the concept that HBoV is a respiratory pathogen.

In agreement with previous studies, co-infection with other pathogens was common in HBoV-positive patients in our study, but no significant differences were found in term of frequencies of specific respiratory symptoms, duration of hospitalization, wheezing and the cytokine production in patients with HBoV infection only and patients with co-infection with other viruses or bacteria. Clearly, our results indicated potential co-infections will not influence clinical outcome of HBoV infection in respiratory tract. This finding strongly suggested the pathogenic potential of HBoV in young children with LRTI.

Because virus co-infection would not increase illness duration or severity in virus-induced respiratory disease in our study or the others [20], [31]–[34]. Therefore the more severe lower respiratory tract symptom presented in high HBoV viral load patients may solely depend on HBoV viral load. In our study, HBoV viral load did not show significant influence on the presentations of upper respiratory tract infection symptoms, such as cough, rhinorrhea, however high HBoV viral load led to more severe lower respiratory tract symptoms and longer hospitalization. It was also indicated *in vitro* studies that viral infection resulted in the high IL-8 level and other pro-inflammatory molecules; these cytokines were likely to bring in additional neutrophils and also to cause hyper-responsiveness in bronchus, which could contribute to the severity of both upper and lower respiratory symptoms during the viral infection. In our study, the IL-8 level was significantly higher in patients with high HBoV viral loads that those who with low viral load. Overall, our findings indicated that the high HBoV viral loads played an important role in the severity of LRTIs, the symptoms and the duration of hospital stay.

No previous studies showed the distinctive clinical signs that help to differentiate HBoV-positive infections from other viral infections [35], [36]. In our study, main clinical symptoms in HBoV-positive patients included cough (100%), wheezing (90.32%), and fever (41.94%). Although the existence of HBoV was not directly associated with the illness, some studies showed wheezing was the main manifestation of HBoV infection [2], [4], [37]–[39]. Our data was consistent with the findings in these studies and support the fact that wheezing was one of the most common symptoms presented in HBoV- positive patients. We also investigated the relationship between HBoV viral load and duration of wheezing, we found that the days of wheezing correlated with viral load. Emily et al documented that persistent HBoV shedding, may increase the duration of respiratory symptoms [6]. This report may explain the link between HBoV load and persistent wheezing.

Because our study is a comprehensive clinical observation on HBoV- positive patients with severe LRTIs, failure of follow-up was one of the limitations to our study, which may led to the loss of some important data, such as the impact of persistent viral shedding on the infection prognosis during the acute viral infection. It is also important to assess the asthma development in these cases if possible.
